# Evaluation of the bacterial diversity of Pressure ulcers using bTEFAP pyrosequencing

**DOI:** 10.1186/1755-8794-3-41

**Published:** 2010-09-21

**Authors:** Drake M Smith, David E Snow, Eric Rees, Ann M Zischkau, J Delton Hanson, Randall D Wolcott, Yan Sun, Jennifer White, Shashi Kumar, Scot E Dowd

**Affiliations:** 1 Research and Testing Laboratory, Lubbock, TX 79407, USA; 2 Southwest Regional Wound Care Center, Lubbock, TX 79410, USA; 3 Medical Biofilm Research Institute, Lubbock, TX 79407, USA

## Abstract

**Background:**

Decubitus ulcers, also known as bedsores or pressure ulcers, affect millions of hospitalized patients each year. The microflora of chronic wounds such as ulcers most commonly exist in the biofilm phenotype and have been known to significantly impair normal healing trajectories.

**Methods:**

Bacterial tag-encoded FLX amplicon pyrosequencing (bTEFAP), a universal bacterial identification method, was used to identify bacterial populations in 49 decubitus ulcers. Diversity estimators were utilized and wound community compositions analyzed in relation to metadata such as Age, race, gender, and comorbidities.

**Results:**

Decubitus ulcers are shown to be polymicrobial in nature with no single bacterium exclusively colonizing the wounds. The microbial community among such ulcers is highly variable. While there are between 3 and 10 primary populations in each wound there can be hundreds of different species present many of which are in trace amounts. There is no clearly significant differences in the microbial ecology of decubitus ulcer in relation to metadata except when considering diabetes. The microbial populations and composition in the decubitus ulcers of diabetics may be significantly different from the communities in non-diabetics.

**Conclusions:**

Based upon the continued elucidation of chronic wound bioburdens as polymicrobial infections, it is recommended that, in addition to traditional biofilm-based wound care strategies, an antimicrobial/antibiofilm treatment program can be tailored to each patient's respective wound microflora.

## Background

An estimated 2.5 million hospitalized Americans currently suffer from decubitus ulcers, also known as bedsores or pressure ulcers,[1] and the annual cost for the prevention and treatment of decubitus ulcers is approximately $10 billion [2]. Chronic wounds such as ulcers have troubled the medical community for centuries, as the first known decubitus ulcer was detected during the autopsy of an Egyptian mummy [3]. Today, approximately 20% of long-term care patients are affected [2].

Decubitus ulcers often result from both external and internal patient factors [4]. Pressure, friction, shear force, and moisture are controllable external factors that affect a patient's susceptibility to decubitus ulcers [5-7]. Internal patient factors such as fever, malnutrition, endothelial dysfunction, and anemia also contribute to a patients susceptibility to ulceration [6,8]. Although exceptional care may alleviate external ulcer promoting factors, skin integrity is largely dependent on overall patient health. The dermis and epidermis rely on other organ systems for nutrition and immune function which when compromised by poor health considerably increase the risk of ulceration [9]. As reviewed elsewhere, those typically most at risk of developing decubitus ulcers are the aged, debilitated, paralyzed, unconscious, or patients with incontinentia pigmenti [4,6,10-12]. Less typical, though of equal concern, are children with similar compromising health conditions [13-15].

Decubitus ulcers are most commonly found on the lower half of the body along bony prominences such as the sacrum and heels of bedridden patients [4,16]. Blood flow to compressed tissue becomes restricted and over time nutrient distribution comes to a relative halt while toxic metabolites begin to accumulate, causing cell death [4,17-19]. Additionally, because circulation is restricted, the patient's immunological response in the vicinity of the wound becomes ineffective and the ability to heal is compromised [8].

The microbiota of chronic wounds is known to play a significant role in the hindrance of wound healing, even in the absence of inflammation. This microbial bioburden exists in wounds predominantly as a biofilm [8,10,20-28]. The onset of decubitus ulcers occurs as described, and it is logical to suggest that this impaired host environment is extremely susceptible to biofilm incursion [29]. Functionally equivalent pathogroups (FEPs) are symbiotic colonies of otherwise nonpathogenic species that act synergistically to promote their own survival at the expense of the host. These FEPs colonize and exist as a cohabitation of many bacterial species, known as a chronic wound pathogenic biofilm (CWPB). It is widely accepted that CWPBs are the primary infectious agent in chronic wounds [20,22,25-32].

Current research efforts have exposed CWPBs as being extremely resistant to antimicrobial therapy and as highly adaptable systems with complex ecologies [21,33]. The use of universal 16 S rRNA amplification and sequencing molecular methods, specifically bacterial tag-encoded FLX amplicon pyrosequencing (bTEFAP), have uncovered many FEPs which comprise CWPBs [22,23,33-39]. As the diversity of these CWPB communities increases, the effectiveness of the host's immune system seems to decrease, leading to the effects on wound healing discussed previously [32].

Several helpful reviews have recently been published summarizing efforts within the scientific community to better understand the microbiology associated with chronic wounds, the resistance these wounds develop as a function of CWPBs, and improvements in the effective use of antibiotics and other treatments on wound healing [21,40]. Also evident in the literature are recent efforts to survey the microbial diversity of venous leg ulcers [41]. However, other work indicates significant differences among pathogens in various wound types, and a survey of decubitus ulcers is a necessary contribution to this body of work [37]. Therefore, the aim of this work is to shed new light on the polymicrobial diversity of chronic decubitus ulcer biofilm infections using bacterial tag-encoded FLX amplicon pyrosequencing.

## Results

The diversity of 49 individual decubitus ulcers was evaluated using the bTEFAP methodology. A total of 225,937 individual sequences longer than 350 bp were analyzed among the 49 samples with 210,836 sequences generating BLASTn hits against the bacterial database and an average sequence identity of 96.7%. A total of 83,705 sequences (39.7%) had identity below 96.5%. Only 2,000 of the total number of analyzed sequences fell below 80% identity. A traceback analysis based upon the divergence of sequences from well described and type sequences was performed.

**Figure 1:**
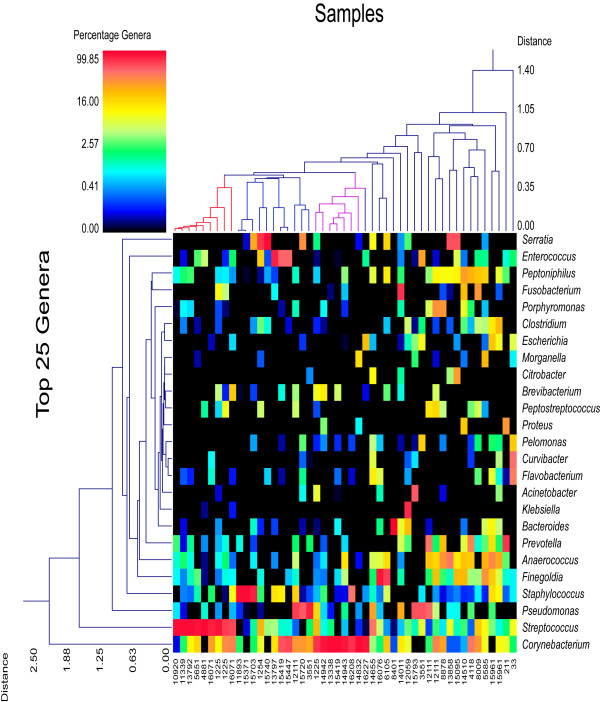
**Double dendogram of major genera in decubitus ulcers**. Describes major genera detected among the 49 samples. The heat map indicates the relative percentage of the given genera within each sample ID with a color legend and scale provided. The distance of the samples based upon weighted pair linkage and Manhattan distance methods with no scaling is provided at the top of the figure along with a distance score. The bacterial genera and the associated clustering are provided along the Y-axis and their associated distance scores indicated.

**Figure 2:**
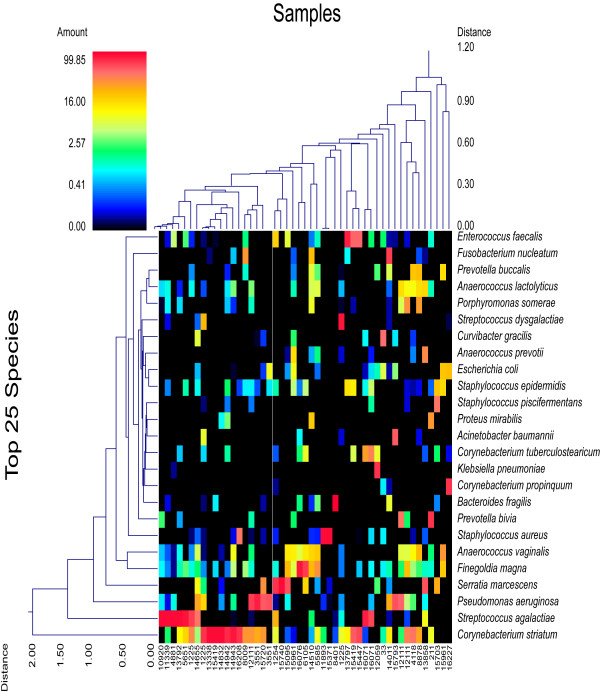
**Double dendogram of major species in decubitus ulcers**. Describes major species detected among the 49 samples. The heat map indicates the relative percentage of the given species within each sample ID with a color legend and scale provided. The distance of the samples based upon weighted pair linkage and Manhattan distance methods with no scaling is provided at the top of the figure along with a distance score. The bacterial species and the associated clustering are provided along the Y-axis and their associated distance scores indicated.

**Figure 3:**
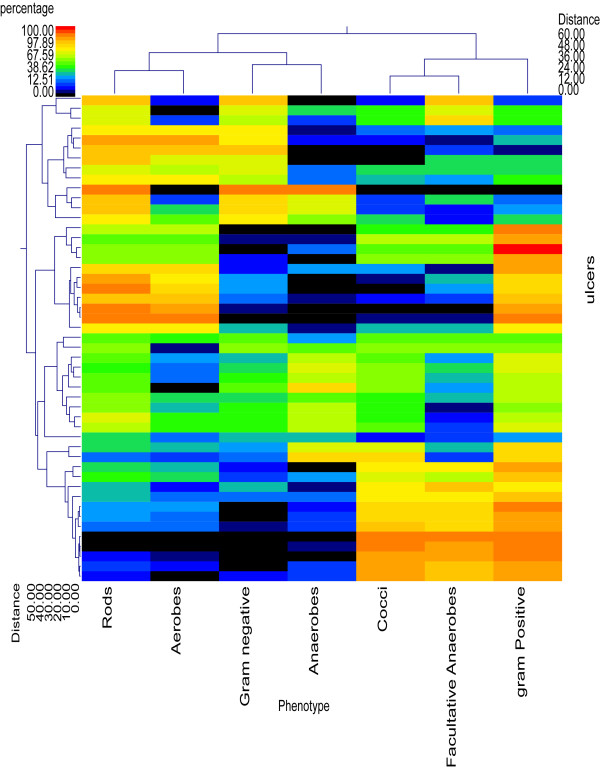
**Double dendogram of phenotypes in decubitus ulcers**. The heat map shows relative percentages of the given phenotypes in each of the 49 samples with a color legend and scale provided. The distance of the samples based upon weighted pair linkage and Manhattan distance methods with no scaling is displayed at the top of the figure along with a distance score. The bacterial phenotypes and the associated clustering are provided along the Y-axis and their associated distance scores are indicated. The bacteria predominantly expressed the gram-positive and cocci phenotypes, with aerotolerance being of relatively equitable distribution.

A total of 212 genera and 487 predicted species (occurring in at least 2 of the wounds) were identified among the 49 wounds. The top 25 unique and most ubiquitous genera (or closest taxonomic designation) are indicated in Table 1 and the top 25 species occurring in the wounds are shown in Table 2. Several genera and species were found in high percentage in individual wounds (Figure 1 and Figure 2). *Corynebacterium striatum *predominated in 12 of the wounds; *Streptococcus agalactiae *predominated in 9 wounds; *Pseudomonas aeruginosa *predominated in 6 wounds; *Anaerococcus vaginalis *and *Anaerococcus prevotii *predominated in 1 wound each, and a mixture of these two species with *Anaerococcus lactolyticus *led to a predominance of this genus in 3 additional wounds; *Serratia marcescens *predominated in 4 wounds; *Staphylococcus aureus *predominated in 3 wounds and a mixture of *Staphylococcus piscifermentans *and *Staphylococcus epidermidis *led to a predominance of this genus in a fourth wound; *Enterococcus faecalis *predominated in 3 wounds; *Prevotella bivida *predominated in 2 wounds and a mixture of *Prevotella bivida *with *Prevotella buccalis *led to a predominance of this genus in a third wound; *Finegoldia magna*, *Fusobacterium nucleatum*, and *Porphyromonas somerae *were predominant in 2 wounds each; and *Bacteroides fragilis*, *Klebsiella pneumoniae*, *Acinetobacter baumannii*, *Curvibacter gracilis*, and *Proteus mirabilis *were each predominant in 1 wound. The remaining wounds were highly diverse with no significantly predominant populations. From the data we note that 69% were gram positive, 31% were anaerobes, 43% were facultative anaerobes, and 69% were rod shaped bacteria (Figure 3). It should also be noted that genera appearing in less than 3 individual samples and also having only low relative percentages < 1% were omitted from this figure to improve readability.

**Table 1:** Evaluation of primary genera among the 49 decubitus ulcer samples

ID	No. of samples	Ave %	Std Dev	Max%	Gram Stain*	Oxygen Tolerance†	Morphology (shape)
*Streptococcus*	45	19.0	33.65	97.5	+	Facultative anaerobe	Cocci

*Corynebacterium*	44	24.8	31.7	99.3	+	Aerobe	Rod

*Staphylococcus*	39	9.2	25.0	99.7	+	Facultative anaerobe	Cocci

*Finegoldia*	32	7.3	18.1	83.5	+	Anaerobe	Cocci

*Anaerococcus*	29	6.6	9.1	36.0	+	Anaerobe	Cocci

*Pseudomonas*	27	14.1	23.7	82.0	-	Aerobe	Rod

*Peptoniphilus*	27	3.6	5.1	19.4	+	Anaerobe	Cocci

*Enterococcus*	24	8.5	19.5	79.9	+	Facultative anaerobe	Cocci

*Prevotella*	24	7.0	16.0	69.0	-	Anaerobe	Rod

*Clostridium*	21	1.49	3.4	14.5	+	Anaerobe	Rod

*Pelomonas*	18	1.5	3.4	11.1	-	Aerobe	Rod

*Bacteroides*	17	7.5	24.0	99.9	-	Anaerobe	Rod

*Flavobacterium*	17	2.8	7.4	30.8	-	Aerobe	Bacillus or coccobacillus

*Porphyromonas*	16	3.4	7.5	23.5	-	Anaerobe	Rod

*Serratia*	15	21.1	30.0	94.9	-	Facultative anaerobe	Rod

*Escherichia*	15	2.5	4.14	12.2	-	Facultative anaerobe	Bacillus or coccobacillus

*Brevibacterium*	14	2.2	3.1	10.8	+	Aerobe	Rod

*Eubacterium*	14	1.3	2.1	6.2	+	Anaerobe	Rod

*Arthrobacter*	14	0.3	0.3	1.0	-	Aerobe	Rod

*Peptostreptococcus*	12	2.6	2.9	9.1	+	Anaerobe	Coccus

*Helococcus*	12	1.3	2.7	9.8	+	Facultative anaerobe	Cocci

*Fusobacterium*	11	9.6	19.4	63.9	-	Anaerobe	Rod

*Dermabacter*	11	0.3	0.3	0.9	+	Facultative anaerobe	Rod

*Sulfurospirillum*	10	1.0	2.0	6.2	-	Aerobe	Rod

*Dialister*	10	0.6	1.0	3.2	-	Anaerobe	Rod

The primary identification based upon percent sequence identity as described in the materials and methods is indicated. The number of samples in which each bacteria was identified is provided along with the average percent (avg %) among the positive samples, the standard deviation (st dev) and the range of percentages among the positive samples. No. of samples = the no. of samples in which the genus were identified Mean % = the average percentage of that genus in each of the samples SD = standard deviation of these percentages Maximum % = the maximum percentage of the genus in each sample * + Gram-positive; - Gram-negative

† Anaerobes are unable to propogate in laboratory media in the presence of oxygen; facultative anaerobes can grow both in the presence and absence of oxygen; aerobes can grow in the presence of oxygen

**Table 2:** Evaluation of primary species among the 49 decubitus ulcer samples

ID	No. of samples	Ave %	Std Dev	Max %	Gram Stain	Oxygen Tolerance	Morphology (shape)
*Corynebacterium striatum*	35	26.1	32.1	99.3	+	Facultative anaerobe	Bacillus or coccobacillus

*Finegoldia magna*	32	7.3	18.1	83.5	+	Anaerobe	Coccus

*Staphylococcus epidermidis*	24	1.8	3.3	12.1	+	Facultative anaerobe	Coccus

*Anaerococcus vaginalis*	23	4.3	5.6	22.8	+	Anaerobe	Coccus

*Pseudomonas aeruginosa*	23	16.5	24.9	82.0	-	Aerobe	Bacillus or coccobacillus

*Streptococcus mitis*	22	0.6	1.9	8.9	+	Facultative anaerobe	Coccus

*Streptococcus parasanguinis*	21	0.2	0.2	0.8	+	Facultative anaerobe	Coccus

*Enterococcus faecalis*	21	8.7	20.6	79.9	+	Facultative anaerobe	Coccus

*Anaerococcus lactolyticus*	20	2.2	3.0	8.5	+	Anaerobe	Coccus

*Peptoniphilus indolicus*	20	1.6	2.4	8.6	+	Anaerobe	Coccus

*Streptococcus agalactiae*	19	37.7	41.6	97.2	+	Facultative anaerobe	Coccus

*Peptoniphilus harei*	18	0.6	1.2	4.3	+	Anaerobe	Coccus

*Pelomonas saccharophila*	18	1.5	3.4	11.1	-	Aerobe	Rod

*Staphylococcus aureus*	17	13.7	33.2	99.7	+	Facultative anaerobe	Coccus

*Peptoniphilus ivorii*	17	1.9	3.2	11.0	+	Anaerobe	Coccus

*Streptococcus thermophilus*	16	0.3	0.5	2.0	+	Facultative anaerobe	Coccus

*Streptococcus constellatus*	16	0.3	0.4	1.6	+	Facultative anaerobe	Coccus

*Escherichia coli*	15	2.5	4.1	12.2	-	Facultative anaerobe	Bacillus or coccobacillus

*Flavobacterium succinicans*	15	0.9	1.2	3.6	-	Aerobe	Bacillus or coccobacillus

*Serratia marcescens*	15	21.1	29.9	94.9	-	Facultative anaerobe	Bacillus or coccobacillus

*Corynebacterium tuberculostearicum*	14	4.4	8.7	27.9	+	Facultative anaerobe	Bacillus or coccobacillus

*Peptoniphilus lacrimalis*	13	1.0	1.8	6.2	+	Anaerobe	Coccus

*Porphyromonas somerae*	13	4.0	8.1	23.3	-	Anaerobe	Bacillus or coccobacillus

*Prevotella buccalis*	12	3.4	4.6	12.7	-	Anaerobe	Bacillus or coccobacillus

Brevibacterium antiquum	12	2.4	3.1	10.8	+	Aerobic	Rod

The primary identification based upon percent sequence identity as described in the materials and methods is indicated. The number of samples in which each bacteria was identified is provided along with the average percent (avg %) among the positive samples, the standard deviation (std dev) and the range of percentages among the positive samples. No. of samples = the no. of samples in which the species were identified Mean % = the average percentage of that species in each of the samples SD = standard deviation of these percentages Maximum % = the maximum percentage of the species in each sample * + Gram-positive; - Gram-negative

† Anaerobes are unable to propagate in laboratory media in the presence of oxygen; facultative anaerobes can grow both in the presence and absence of oxygen; aerobes can grow in the presence of oxygen

**Figure 4:**
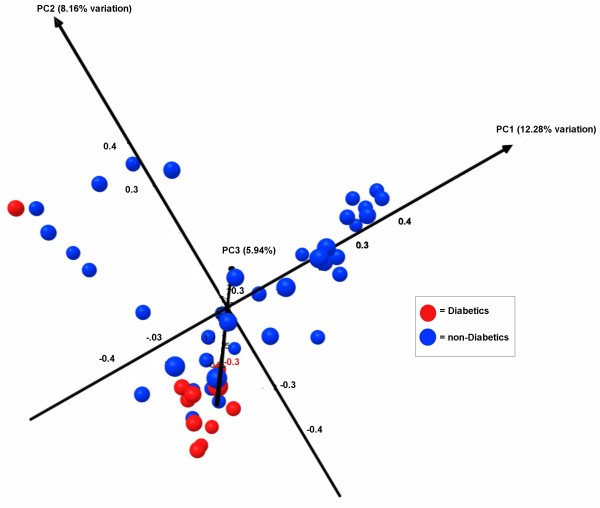
**Three dimensional PCA plot of the unifrac distance in relation to diabetes**. Principle component analysis based upon a unifrac analysis of the sequencing data was utilized. Based upon this PCA analysis the primary 3 vectors are plotted in 3 dimensions. The percent variability explained by each vector is indicated in parenthesis in the figure. Based upon the primary vector a t-test was utilized to determine if there was significant variation between diabetic samples. The separation across the primary vector was found to be significant (p = 0.003) indicating that the community structure of ulcers in subjects with diabetics may be different from those of non-diabetics.

We further evaluated microbial diversity in relation to metadata that included Age group, Gender, Race, diabetes, vascular or coronary co-morbidities, paraplegia or quadriplegia, and time it took to heal the wound. There were no significant difference or clustering of the microbial diversity observed in relation to any of the metadata except diabetes (Figure 4). Based upon the PCA1 loading and two tailed t-test the separation explained by the primary vector was significant for microbial diversity in the ulcers depending on whether the patient had diabetes (p = 0.003). Measures of diversity analyses were evaluated using Rarefaction, Shannon index, Ace, and Chao1 at the 3% and 5% divergence levels (corresponding to species and genus respectively). An average of 3964 sequences per sample were evaluated with summary data on average diversity measurements evaluated statistically using Mann-Whitney and Kruskal-Wallis tests in relation to the metadata. No significant differences were observed for diversity measures in relation to metadata parameters even antibiotic usage.

## Discussion

The results indicate that there was a considerably large diversity in the samples, with 228 identified genera and 487 identified species among 49 decubitus ulcers. Additionally, the 79,837 sequences with identity less than 96.5% can be considered as previously unknown or uncharacterized species of bacteria [42]. The majority of these organisms were most closely related to *Staphylococcus*, *Enterococcus*, *Serratia*, *Pseudomonas*, *Streptococcus*, and *Corynebacterium *spp based upon 16 S sequence. These bacteria were classified based upon their closest identification and ranked at the most suitable genus, family or order level.

Our data indicates a number of important FEPs associated with decubitus ulcers (Figure 1 and Figure 2 dendograms). At a relative distance of 0.30 based upon the weighted-pair linkage and Manhattan distance in Figure 2 we note there are 8 primary clusters, which included 8 predominant groups representing possible pathogroups. It is also evident that *Staphylococcus aureus*, *Corynebacterium striatum*, *Streptococcus agalactiae*, *Serratia marcescens*, *Finegoldia magna*, *Pseudomonas aeruginosa*, and *Enterococcus faecalis *are defining variables for these 8 clusters, with *Streptococcus agalactiae *being the defining variable for two separate clusters. It is interesting that so many of these wounds were predominated by what are either facultative or obligate anaerobic bacteria with only very minor populations of aerobes (36%). This suggests that such anaerobes might be contributing significantly to the etiology of chronic wound biofilm infections.

Most of the wounds we have evaluated contain relatively high overall numbers of bacteria (> 10^5 ^per mg debridement), based upon quantitative molecular methods so even ostensibly low percentages of individual species may potentially represent a large number of individual bacteria that have colonized or been recruited into and commenced propagating within decubitus ulcer biofilms even if we are considering populations that represent only 1% of the total. It is also important to note that, in addition to chronic wounds such as decubitus ulcers, healthy human skin is host to a diverse community of microbes.

Several recent surveys have shown that intra- and inter-personal diversity of the human skin microbiome is highly variable depending on gender, body regions, handedness, and time [43-46]. Skin microflora are found to fluctuate greatly in a short period of time, [43] women are shown to have a higher diversity than men, the dominant hand is shown to be more diverse than its counterpart, and skin regions are shown to be more similar in microbial composition with greater proximity [44]. In addition, the genera *Corynebacterium*, *Propionibacteria*, and *Staphylococcus *have been identified as primary constituents of healthy skin microflora, with *Corynebacterium *and *Staphylococcus *predominating the moist environments [45]. It is no surprise, then, to have detected *Corynebacterium *and *Staphylococcus *in 44 and 39 respective wounds out of 49 surveyed. These ubiquitous skin bacteria may sometimes be the primary, opportunistic pathogenic constituents of CWPBs, as seen in Figures 1 and 2.

Resident skin microflora may be considered largely commensal in nature, but it is known that nonpathogenic microbes often become opportunistically pathogenic when the skin barrier becomes impaired [47]. The difference between a pathogenic dermatophyte and a resident skin bacterium is commonly defined not only by the inherent properties of the organism, but also by the host's ability to resist infection. Resident skin bacteria are capable of expressing virulence factors which under certain circumstances may allow for the evasion of host defensive onslaught, but the primary determinant of pathogenicity lies in the effectiveness of host response to microbial invasion [48]. It may be that conditions within the open wound environment allow for much greater propagation of resident cutaneous microflora, which colonize the wound without substantial opposition. In some cases, these nonpathogenic skin residents may harbor pathogenic recruits by concealing antigenic factors, thus preventing recognition by the host's primary immune cells.

The 49 sampled wounds were found to be sufficiently diverse in composition as to negate any generalized recommendations for the targeting of microbial bioburdens within chronic wounds. Instead, each wound is shown to be exceptionally unique, with some wounds predominated by opportunistically pathogenic commensal skin residents and others predominated by known pathogens. Other wounds showed no clear predomination of a single species but rather a heterogeneous and complex assemblage. In addition, the present study is incapable of determining the role of certain bacteria that have been detected in lower, albeit potentially significant percentages. Even bacteria which comprise approximately 15% of a wound, as indicated in Figures 1 and 2, may contribute to the etiology of chronic wounds as this percentage represents a substantial number of bacteria even in a wound with only moderate bioburden at critical colonization levels. It is therefore recommended that future treatment courses be customized for each individual wound due to the highly variable etiology of chronic wounds such as decubitus ulcers.

Taking into consideration the metadata we did not find any significant differences in ecological measurements of microbial diversity or population richness (rarefaction, ace, Chao1) we did notice a significant difference in ulcers associated with their community composition in relation to whether the patient had diabetes or not. This suggests that the types of pathogens and opportunistic pathogens that populate pressure ulcers may be significantly different if the subject also has diabetes. Diversity measurements suggest that up to an average of 337 species and 180 genera of bacteria may be present in a pressure wound. These numbers can be misleading on two levels. First further optimization of next generation data processing will enhance the reliability of such measurements from a technical standpoint. Second, it is well understood that many of the bacteria that may be present in chronic wounds may not be active participants of the community of such bioburdens but may transient populations or environmental contaminants. These large numbers are also strongly influenced by the very low relative abundance populations, which for the sake of discussion might be considered populations occurring at < 0.1% in wounds, yet their numbers still contribute to such diversity estimates. We also observed a relatively high standard deviation in relation to these diversity estimators suggesting that there is a broad range of diversity among pressure ulcers. In addition although we took every preprocessing precaution to enable the most accurate diversity estimation and modeling, newer methods must certainly be tested and validated to provide more accurate descriptions of the microbial diversity in environments such as wounds.

Diversity measurements (Table 3) coordinated with the community structures represented in Figures 1 and Figure 2 illustrate that in most ulcers there are a handful primary pathogens and opportunistic pathogens that make up the largest components of these communities. Thus, chronic wounds are highly polymicrobial and predominant populations (e.g. > 0.1% of total proportion) must be considered clinically as potential opportunistic players in such infections. To ignore even a potential role of such microorganisms is also to limit the potential efficacy of any type of therapy that is targeting the microbial bioburden/biofilm. If we target only the primary population then ecological principles of selection will dictate that minor populations will succeed into this, now available, niche. If we can find a combination of treatments (e.g. antibiotic, antibiofilm, or antimicrobial) that can target all of the primary or predominant populations in a wound then we have introduced a potentially larger barrier that the very minor populations must overcome to establish their own niche. Even as new populations potentially integrate into the bioburden a new round of diagnostics and a new targeted therapy can be implemented. This becomes a very easy cycle of diagnose and treat that is the cornerstone of medicine. To further the case and point recent diagnostic developments in relation to chronic wounds and their polymicrobial bioburdens, have already been shown to provide definitive and significant improvement in both healing rate and outcome [20]. Now taking this comprehensive diagnostics to the next level, targeted and personalized therapies directed by these diagnostics are currently available. Clinical effectiveness studies are currently in final stages and already clearly show how personalized treatments directed by comprehensive molecular diagnostics may change the way wounds are treated thereby improving the lives of those afflicted and saving millions if not billions in health care dollars.

**Table 3:** Summary of diversity estimators Rarefaction, Shannon index, Ace, and Chao1

Variable	Observations	Minimum	Maximum	Mean	Std. deviation
Rarefaction 3%	49	117.000	601.000	337.898	107.599
Rarefaction 5%	49	41.000	327.000	180.082	71.995
Shannon 3%	49	2.896	5.614	4.703	0.670
Shannon 5%	49	2.353	5.166	3.955	0.763
OTU 3%	49	117.000	601.000	337.898	107.599
OTU 5%	49	41.000	327.000	180.082	71.995
ACE 3%	49	181.962	911.814	446.255	159.805
ACE 5%	49	72.165	515.946	235.668	102.497
Chao1 3%	49	189.571	733.412	436.274	145.754
Chao1 5%	49	72.000	412.263	228.350	94.776

Diversity metrics related to 3% and 5% divergence (species and genera levels respectively) are provided along with basic summary statistics. These include the minimum values across all samples, the maximum, the mean and the standard deviation.

## Conclusion

The present study is a continuation of the research studies conducted by Dowd et al. and Wolcott et al. [29,30,35,37] using bTEFAP to survey the microbial ecology of diabetic foot ulcers, venous leg ulcers, surgical site infections, and decubitus ulcers. The overwhelming outcome of these collective studies is that traditional culture techniques are still largely inadequate in determining the microbial composition of many chronic wounds. Chronic infections such as Venous, Diabetic extremity, and Pressure ulcers, have proven to be much more of a diverse and complicated microbial ecology than previously recognized. Most interestingly, obligate anaerobes were found to be a significant proportion, if not a clear majority in chronic wounds, in all surveyed wound types. Requiring specialized collection, transport, and analysis methods for culture based diagnostics of anaerobes will remain difficult especially as part of polymicrobial infections. We demonstrate that chronic wounds such as decubitus ulcers are not merely infected by a single pathogenic species of bacteria, but rather by a blending of symbiotic microbes which form a CWPB. Whether termed bioburden or biofilm, the microbial participation in chronic wounds have been shown to represent a major contributing factor to the resistance of natural healing in chronic wounds, [37,49,50] and the great diversity of these microbial communities adds to their resilience against traditional host and medicinal onslaughts [32]. Previous efforts along with the present study indicate that the highly unique profile of each individual wound would require a therapeutic approach specifically tailored to the patient's respective wound microflora in addition to the multitude of procedures already implemented in the assuage of chronic wounds and support of the host and comorbidities [51,52].

## Methods

### General sample collection methods

Patients were identified with decubitus ulcers and then enrolled in the study protocol after being educated and signing the informed consent protocol in compliance with Western Institutional Review Board approved protocols 56-RW-004 WIRB^® ^Protocol #20062347. All necessary details were thoroughly explained to the patients and written consent was obtained in the presence of a third party witness. A copy of the consent form has been provided to the journal editors. Patients were knowledgeable of their right to discontinue participation at any time despite the established written consent. Samples from decubitus ulcer wound-beds were collected using sharp debridement as a regimen of standard care with aseptic precautions. Samples were placed into sterile 2 ml eppendorf tubes and frozen at -80°C. For metadata analysis we utilized age groups 20 (20-40), 50 (41-60), 80 (61 and older), presence of vascular or heart problems, diabetes, systemic antibiotic use, number of days with wounds at time of evaluation (arbitrarily grouped into <100 days, 100-900, and 901+ days), presence or absence of any type of immobility (e.g. paraplegia or quadriplegia), race, and gender.

### DNA Extraction

After thawing, the debridement samples were centrifuged at 14,000 rpm for 30 seconds and resuspended in 500 μl RLT buffer (Qiagen, Valencia, CA) (with β-mercaptoethanol). A sterile 5 mm steel bead (Qiagen, Valencia, CA) and 500 μl sterile 0.1 mm glass beads (Scientific Industries, Inc., NY, USA) were added for complete bacterial lyses in a Qiagen TissueLyser (Qiagen, Valencia, CA), run at 30 Hz for 5 min. Samples were centrifuged briefly and 100 μl of 100% ethanol added to a 100 μl aliquot of the sample supernatant. This mixture was added to a DNA spin column, and DNA recovery protocols were followed as instructed in the QIAamp DNA Mini Kit (Qiagen, Valencia, CA) starting at step 5 of the Tissue Protocol. DNA was eluted from the column with 30 μl water and samples were diluted accordingly to a final concentration of 20 ng/μl. DNA samples were quantified using a Nanodrop spectrophotometer (Nyxor Biotech, Paris, France).

### Massively parallel bTEFAP and bTEFAP titanium

Bacterial tag-encoded FLX amplicon pyrosequencing (bTEFAP) was performed as described previously [37]. The new bacterial tag-encoded FLX-Titanium amplicon pyrosequencing (bTEFAP) approach is based upon similar principles to the previous generation of bTEFAP but utilizes Titanium reagents and titanium procedures and a one-step PCR, mixture of Hot Start and HotStar high fidelity taq polymerases, and amplicons originating from the 27F region numbered in relation to *E. coli *rRNA. The bTEFAP procedures were performed at the Research and Testing Laboratory (RTL; Lubbock, TX) based upon RTL protocols http://www.researchandtesting.com.

### Bacterial diversity data analysis

Following sequencing, all failed sequence reads, low quality sequence ends and tags were removed. Sequences were depleted of any non-bacterial ribosome sequences and definite chimeras using custom software described previously [53] and the Black Box Chimera Check software B2C2 (described and freely available at http://www.researchandtesting.com/B2C2.html. Sequences less than 350 bp (bTEFAP titanium) were removed. The identities of decubitus ulcer sequences were determined by first using a distributed BLASTn .NET algorithm [54] against a database of high quality 16 s bacterial sequences derived from NCBI. Database sequences were characterized as high quality based upon the criteria of RDP ver 9 [55] Using a .NET and C# analysis pipeline the resulting BLASTn outputs were compiled, validated using taxonomic distance alignment methods, and data reduction analysis performed as described previously [30,37,56]. Rarefaction, ace and Chao1 to estimate maximum diversity in wound using of 300 bp trimmed, non-ribosomal sequence depleted, chimera depleted, high quality reads was performed as described previously [53,56,29,30] using Mothur [57-59].

### Bacterial identification

The bacteria were classified at the appropriate taxonomic levels based upon the above BLASTn derived sequence identity (percent of total length query sequence which aligns with a given database sequence) and validated using taxonomic distance methods. Sequences with identity scores, to known or well characterized 16 S sequences, greater than 97% identity (< 3% divergence) were resolved to the species, between 95% and 97% to the genus, between 90% and 95% to the family and between 80% and 90% to the order level etc. After resolving based upon these parameters, the percentage of each bacterial ID was individually analyzed for each wound providing relative abundance information within and among the decubitus ulcers based upon relative numbers of reads within a given sample. Evaluations presented at a given taxonomic level, except specific level, represent all sequences resolved to their primary generic identification or their closest relative (where indicated).

### Basic Statistics

Statistics were performed using comparative functions and multivariate hierarchical clustering methods of NCSS 2007 (NCSS, Kaysville, Utah). Using methods described previously, a Unifrac-based principle component analysis (PCA) was utilized to determine significant relationships in the microbial population of ulcers based upon metadata. In short sequences were trimmed to include a uniform length of 350 assembled at 99% similarity using NGEN (DNAstar, Madison WI), alignment of unique sequences performed with Muscle [58], distance matrix created using DNAdist algorithm of the Phylip package [59], and PCA analysis performed in R (R Development Core Team). Other basic statistics were performed in excel and with XLStat (Addinsoft, NY)

## Competing interests

SED and RDW are owners of Research and Testing Laboratory which provides commercial and research services related to bTEFAP. SED and RDW are owners of Pathogenius Diagnostics which is a clinical diagnostic company with a specific focus on diagnosis of chronic wound infections. SED and RDW have patents submitted on molecular pathogen diagnostic methods. All other authors are employees of either Pathogenius Laboratory or Research and Testing Laboratory. SK was a student worker with no formal ties any no competing interests.

## Authors' contributions

DMS, DS, JDH prepared and edited manuscript, AMZ, YS, JW performed laboratory methods, RDW obtained clinical samples, SK assisted with data analysis, SED conceived of project, developed molecular and analysis tools, analyzed data, reviewed final versions of manuscripts. All authors have read and approved the final manuscript.

## Pre-publication history

The pre-publication history for this paper can be accessed here:

http://www.biomedcentral.com/1755-8794/3/41/prepub
